# Reduced Dose Intensity of Daunorubicin During Remission Induction for Low-Risk Patients With Acute Lymphoblastic Leukemia: A Retrospective Cohort Study of the Chinese Children’s Cancer Group

**DOI:** 10.3389/fonc.2022.911567

**Published:** 2022-06-07

**Authors:** Yong Zhuang, Kefei Wu, Xiaofan Zhu, Jiaoyang Cai, Shaoyan Hu, Ju Gao, Hua Jiang, Xiaowen Zhai, Xin Tian, Yongjun Fang, Runming Jin, Qun Hu, Hui Jiang, Ningling Wang, Lirong Sun, Wing Kwan Leung, Minghua Yang, Kaili Pan, Xuedong Wu, Changda Liang, Shuhong Shen, Jie Yu, Xiuli Ju

**Affiliations:** ^1^ Department of Pediatrics, Qilu Hospital of Shandong University, Jinan, China; ^2^ National Children’s Medical Center, Department of Hematology/Oncology, Key Laboratory of Pediatric Hematology and Oncology of China Ministry of Health, Shanghai Children’s Medical Center, Shanghai Jiao Tong University School of Medicine, Shanghai, China; ^3^ State Key Laboratory of Experimental Hematology and Division of Pediatric Blood Diseases Center, Institute of Hematology and Blood Diseases Hospital, Peking Union Medical College, Chinese Academy of Medical Sciences, Tianjin, China; ^4^ Department of Hematology/Oncology, Children’s Hospital of Soochow University, Suzhou, China; ^5^ Department of Pediatrics, Key Laboratory of Birth Defects and Related Disease of Women and Children, Ministry of Education, West China Second University Hospital, Sichuan University, Chengdu, China; ^6^ Department of Hematology/Oncology, Guangzhou Women and Children’s Medical Center, Guangzhou, China; ^7^ Department of Hematology/Oncology, Children’s Hospital of Fudan University, Shanghai, China; ^8^ Department of Hematology/Oncology, Kunming Children’s Hospital, Kunming, China; ^9^ Department of Hematology/Oncology, Children’s Hospital of Nanjing Medical University, Nanjing, China; ^10^ Department of Pediatrics, Union Hospital of Tongji Medical College, Huazhong University of Science and Technology, Wuhan, China; ^11^ Department of Pediatrics, Tongji Hospital of Tongji Medical College, Huazhong University of Science and Technology, Wuhan, China; ^12^ Department of Hematology/Oncology, Shanghai Children’s Hospital, Shanghai Jiao Tong University, Shanghai, China; ^13^ Department of Pediatrics, Anhui Medical University Second Affiliated Hospital, Hefei, China; ^14^ Department of Pediatrics, Affiliated Hospital of Qingdao University, Qingdao, China; ^15^ Department of Pediatrics, Hong Kong Children’s Hospital, The Chinese University of Hong Kong, Hong Kong, Hong Kong SAR, China; ^16^ Department of Pediatrics, Xiangya Hospital Central South University, Changsha, China; ^17^ Department of Hematology/Oncology, Xi’an Northwest Women and Children Hospital, Xi’an, China; ^18^ Department of Pediatrics, Nanfang Hospital, Southern Medical University, Guangzhou, China; ^19^ Department of Hematology/Oncology, Jiangxi Provincial Children’s Hospital, Nanchang, China; ^20^ Department of Hematology/Oncology, Chongqing Medical University Affiliated Children’s Hospital, Chongqing, China

**Keywords:** acute lymphoblastic leukemia, children, remission-induction, multicenter study, daunorubicin

## Abstract

It is urgently necessary to reduce the adverse effects of chemotherapy while maintaining their cure high rates for children with acute lymphoblastic leukemia (ALL). The present study aimed to determine whether the dose intensity of daunorubicin during the remission-induction phase could be reduced for low-risk patients with ALL. A total of 2396 eligible patients, who participated in CCCG-ALL-2015 study and were provisionally assigned to the low-risk group, were included and divided into single-dose group and double-dose group according to the dosage of daunorubicin during the remission-induction phase. For patients with *ETV6-RUNX1* positive ALL or hyperdiploidy ALL, there were no significant differences in outcomes between the two groups. For other patients, the 5-year event-free survival rate was significantly better and the 5-year cumulative risk of any relapse was significantly lower in the double-dose group compared with the single-dose group. Both the 5-year overall survival rate and the risk of early deaths were not significantly different between the two groups. Our results suggested that only B-lineage ALL patients with *ETV6-RUNX1* positivity or hyperdiploidy who achieved an early negative minimal residual disease status were suitable candidates for dosage reduction of daunorubicin during the remission-induction phase.

**Clinical Trial Registration:**

http://www.chictr.org.cn/showproj.aspx?proj=10115, identifier ChiCTR-IPR-14005706.

## Introduction

Acute lymphoblastic leukemia (ALL) is the most common childhood hematologic malignancy and was considered to be an intractable disease (1). Fortunately, contemporary risk-directed treatment and improved supportive care have improved 5-year event-free survival and overall survival rates in childhood ALL to over 80% and 90%, respectively, and the cumulative risk of relapse has been decreased to less than 10% in many clinical trials (2–12). However, the associated acute and chronic health disabilities mainly caused by the toxicities of chemotherapeutic drugs have dimmed this success (13). Therefore, current efforts are focused on improving not only the cure rate but also the quality of life of the patients by reducing treatment-related toxicities (14, 15).

Analysis of legacy clinical trials conducted in the 1980s and 1990s shows that approximately 40% to 50% of all children and adolescents with ALL were cured with much less intensive chemotherapy regimens (16–18), but the lack of precise criteria by which to identify patients who could be cured with reduced-intensity regimens has hindered progress in decreasing treatment intensity. On the other hand, the regimens used for patients without high-risk disease features currently are generally much more intensive than those used historically, suggesting that many children with ALL are overtreated (19, 20). In this context, there is a strong rationale for identifying a subset of ALL patients who can be treated with a reduced-intensity regimen.

By retrospectively analyzing the clinical data of the Chinese Children’s Cancer Group (CCCG) -ALL-2015 study, a prospective, multi-institutional clinical trial involving 20 major hospitals and medical centers, this report evaluated the efficacy of single-dose and double-dose of daunorubicin during remission induction for low-risk patients, aiming to determine which subset of ALL patients can be treated with a reduced-intensity regimen.

## Materials and Methods

The chemotherapy protocol of CCCG-ALL-2015 study, a minimal residual disease (MRD)-directed, risk-stratified protocol, was established based on the St. Jude Children’s Research Hospital Total XV and XVI studies (9) and the Shanghai Children’s Medical Center (SCMC) ALL-2005 trial (21). Children aged between 0 to 18 years with a confirmed diagnosis of ALL (secondary leukemia was excluded) were provisionally assigned to the low-risk, intermediate-risk, and high-risk groups based on their clinical features and immunophenotype. The final risk status was determined by the molecular subtype of leukemia and MRD (22). MRD negative was defined as less than 10^-4^ in bone marrow. Our protocol included a central review of MRD testing, periodic internal and on-site monitoring, and external auditing to ensure protocol compliance and appropriate data management. All the participating centers obtained ethical approval from their institutional ethics committee.

### Patients

From January 2015 to December 2017, patients provisionally assigned to the low-risk group were identified from the Study Data Center, and further information about the daunorubicin administration during remission induction was retrospectively collected from 18 participating centers. Written informed consent was obtained from all patients or their legal guardians. The initial stratification of ALL patients was carried out based on the CCCG-ALL-2015 protocol. Specifically, patients with B-lineage ALL (B-ALL), who met one of the following criteria: 1. age ≥ 365 days and < 10 years and presenting leukocyte count <50 × 10^9^/L; or 2. hyperdiploidy (≥ 50 chromosomes or DNA index ≥1.16); or 3. *ETV6-RUNX1* fusion-positive; but not have 1. central nervous system (CNS) leukemia and/or testicular leukemia; and 2. adverse genetic features: t(9;22) or *BCR-ABL1* fusion; t(1;19) or *TCF3-PBX1* fusion; or hypodiploidy (< 44 chromosomes); *iAMP21*, were initially assigned to the low-risk group. Patients with a MRD of 1% or more in bone marrow on day 46 of induction and infants younger than 6 months with *KMT2A* rearrangement and a leucocyte count of 300 × 10⁹/L or more were considered to have high-risk ALL. The remaining participants were classified as having intermediate-risk ALL.

### Data Collection

The following data were retrieved from the Study Data Center, such as patients’ demographic data, leukemia information, risk stratification, and treatment outcome. Additional data were retrospectively collected for each subject from participating centers, including the application time of the second dose of daunorubicin, white blood cell (WBC) counts, and absolute neutrophil counts (ANC) at the time of application.

### Study Design and Treatment

Generally speaking, patients received multi-agent chemotherapy at the following treatment phases: induction (week 1-7), consolidation (week 8-15), continuation therapy and reinduction (week 16-34), and maintenance phase (week 35-125). In the first 7 weeks of treatment, all patients received upfront window therapy with dexamethasone for 4 days, followed by remission induction with prednisone, vincristine, daunorubicin, and pegaspargase from day 5 to day 28, as well as CAM regimens (consist of cyclophosphamide, cytarabine, and mercaptopurine) from day 29 to day 35. Patients with B-ALL who had MRD ≥1% on day 19 of remission induction received additional early intensification therapy with cyclophosphamide, cytarabine, mercaptopurine, vincristine, and pegaspargase (days 50-57). Triple intrathecal therapy was given on days 5, 19, and 29 of induction therapy; additional intrathecal treatments on days 8,12 and 15 were given to patients with traumatic lumbar puncture findings at diagnosis.

Upon completion of induction (between days 46 and 49), consolidation treatment was begun with high-dose methotrexate and triple intrathecal therapy every other week, with daily mercaptopurine for 4 courses. Postremission continuation treatment (weeks 16-53) for patients with low-risk ALL consisted of daily mercaptopurine and weekly methotrexate, interrupted by pulses of dexamethasone plus vincristine with triple intrathecal therapy (every 3-4 weeks) and 2 reinduction treatments. For the patients with intermediate-/high-risk ALL, additional pegaspargase and daunorubicin were given every 3 weeks. Subsequent continuation therapy for patients with low-risk ALL consisted of daily mercaptopurine and weekly methotrexate between weeks 54 and 109, with or without pulse therapy with dexamethasone plus vincristine on week 8. For those in the combined intermediate-/high-risk group, an additional drug pair of cyclophosphamide and cytarabine replaced methotrexate and mercaptopurine every 8 weeks, followed by a rest for 1 week. Treatment concluded with daily mercaptopurine and weekly methotrexate (weeks 110-125).

Early treatment response was evaluated on day 19 with morphologic criteria and flow cytometric MRD measurements. MRD ≥ 1% signified poor early treatment response, and provisional low-risk patients were escalated to intermediate-risk. After CAM chemotherapy, MRD measurement was performed on day 46 for intermediate/high-risk patients or provisional low-risk patients with an MRD > 0.1% on day 19. For day 46 MRD ≥ 1%, provisional low/intermediate-risk patients were escalated to the high-risk group. For day 46 MRD ≥ 0.01%, provisional low-risk patients were escalated to the intermediate-risk group. All patients were supposed to receive one dose of daunorubicin at 25 mg/m² over 1 h on day 5 and day 12 in the induction phase. For patients with WBC < 1.0×10^9^/L or ANC < 0.3×10^9^/L on day 12, the second dose of daunorubicin could be postponed for up to 7 days. After 7 days, if WBC or ANC remained below the criteria, the second dose of daunorubicin could be omitted in the low-risk patients. Therefore, in the low-risk group, some patients received two doses of daunorubicin, and some received only one dose. A comparative analysis, including the 5-year event-free survival rate, the 5-year overall survival rate, the 5-year cumulative risk of any relapse, and the risk of early deaths (death during the remission-induction phase), was performed between these two subgroups.

### Statistical Analysis

Event-free and overall survival curves were estimated using the Kaplan-Meier method and compared using the log-rank test. Event-free survival time was calculated from diagnosis to the first treatment failure, including induction failure owing to death or drug resistance, relapse in any site, death due to any cause, development of a secondary malignant tumor, and the instigation of off-protocol therapy by the decision of the treating physician based on persistent disease, severe toxic effects, or transplant not according to protocol criteria. Overall survival was considered as the time from diagnosis to death due to any cause. The cumulative incidence functions of any relapse, or death due to toxic effects were estimated according to Kalbfleisch and Prentice (23) and compared using the Gray test to account for competing events. Competing events for relapse included death in remission, secondary malignant tumor, treatment abandonment, refusal of protocol treatment (withdrawal consent) by parents, or off-protocol treatment by the decision of the treating physician. Competing events for deaths due to toxic effects included relapse and other events (listed above). We calculated 95% CIs with a large-sample normal approximation. All reported *P* values are 2-sided and not adjusted for multiple comparisons, with *P* < 0.05 indicating significance.

Outcome data reported in this article were updated on September 1, 2020. The median follow-up time was 55.1 months (IQR, 47.8-64.4 months; range, 0.4-84.9 months). All statistical analyses were conducted with R statistical software, version 4.0.2 (R Project for Statistical Computing).

## Results

### Study Population

From January 2015 to December 2017, a total of 2489 patients with newly diagnosed ALL from 18 centers were provisionally assigned to the low-risk group in the CCCG-ALL-2015 study. Of these patients, 53 missed information on the use of the second dose of daunorubicin, and 40 were mis-grouped for final risk (day 19 MRD 0.1-0.99% and day 46 MRD missing, or > 0.01%, should have been in the intermediate/high group, but were still in the low-risk group). Therefore, a total of 2396 children were eventually included in the study. Of the 2396 eligible patients (1 403 males [58.6%] and 993 females [41.4%]; median age, 4.1 [range, 1.1-15.8] years), 600 received one dose of daunorubicin (single-dose group), and 87 of them (14.5%) were classified as at the intermediate/high risk based on the MRD level at the end of induction. Moreover, 1796 patients received two doses of daunorubicin (double-dose group), and 316 of them (17.6%) were eventually assigned to intermediate/high risk. There were no significant differences in the initial treatment response based on the detection of MRD on days 19 and 46 of remission induction or the complete remission rate between single-dose daunorubicin and double-dose daunorubicin treated patients (*P*=0.089). Specifically, of the 600 patients, there were 205 patients with *ETV6-RUNX1*-positive ALL and 185 patients with hyperdiploidy, and 210 patients had neither of the genetic alterations. The complete remission rates of them were 90.7% (186/205), 90.8% (168/185) and 86.7% (182/210), respectively. Of the 1796 patients, there were 526 patients with *ETV6-RUNX1*-positive ALL and 451 patients with hyperdiploidy, and 819 patients had neither of the genetic alterations. The complete remission rates of them were 91.6% (482/526), 87.8% (396/451) and 86.6% (709/819), respectively. Of the 1993 patients eventually assigned to the low-risk group, 1240 patients met the criteria for the second dose of daunorubicin (WBC > 1.0×10^9^/L and ANC > 0.3×10^9^/L), of which 1217 actually received the second dose, while 23 did not. Meanwhile, 753 patients did not meet the criteria, of which 263 received two doses of daunorubicin in the induction phase, while 490 received one dose. The clinical and laboratory characteristics of these patients were shown in **Table 1**.

**Table 1:** Clinical and laboratory characteristics of patients with provisional low-risk ALL.

Characteristic	Final risk-Low risk			Final risk- Intermediate/high risk			All patients
	Single-dose group	Double-dose group	*P* value	Single-dose group	Double-dose group	*P* value	
Age, y							
Median	4.2 (1.1-12.0)	4.7 (1.1-15.8)	0.000	4.1 (1.5-9.3)	4.7 (1.1-11.7)	0.025	4.1(1.1-15.8)
Sex							
Male	295 (57.5%)	872 (58.9%)	0.575	46 (52.9%)	190 (60.1%)	0.224	1403(58.6%)
Female	218 (42.5%)	608 (41.1%)		41 (47.1%)	126 (39.9%)		993(41.4%)
WBC count, ×10^9^/L							
Median	16.4 (0.6-234.7)	11.0 (0.3-237.0)	0.000	11.2 (1.2-57.6)	13.8 (1.0-125.2)	0.172	12.5(0.3-237.0)
Meet the criteria*							
Yes	23 (4.5%)	1217 (82.2%)	0.000	2 (2.3%)	216 (68.4%)	0.000	1458 (60.9%)
No	490 (95.5%)	263 (17.8%)		85 (97.7%)	100 (31.6%)		938 (39.1%)
Molecular or cytogenetic abnormalities							
*ETV6-RUNX1*	189 (36.8%)	467 (31.6%)	0.000	16 (18.4%)	59 (18.7%)	0.478	731 (30.5%)
Hyperdiploidy	154 (30.0%)	359 (24.2%)		31 (35.6%)	92 (29.1%)		636 (26.5%)
B lineage, other	170 (33.2%)	654 (44.2%)		40 (46.0%)	165 (52.2%)		1029 (43.0%)
MRD at d 19, %							
< 0.1	391 (76.2%)	1207 (81.6%)	0.009	0	0	0.464	1598 (66.7%)
0.1~0.99	122 (23.8%)	273 (18.4%)		19 (21.8%)	58 (18.4%)		472 (19.7%)
≥1	0	0		68 (78.2%)	258 (81.6%)		326 (13.6%)
MRD at d 46, %							
<0.01 or not performed	501 (97.7%)	1444 (97.6%)	0.905	38 (43.7%)	151 (47.8%)	0.497	2134 (89.1%)
≥ 0.01	12 (2.3%)	36 (2.4%)		49 (56.3%)	165 (52.2%)		262 (10.9%)

*The criteria for the second dose of daunorubicin (WBC > 1.0×10^9^/L and ANC > 0.3×10^9^/L).

### The Outcomes Are Similar for Final Intermediate/High Risk Patients Who Receive Two Doses of Daunorubicin Compared With Those Receiving One Dose

**Figure 1:**
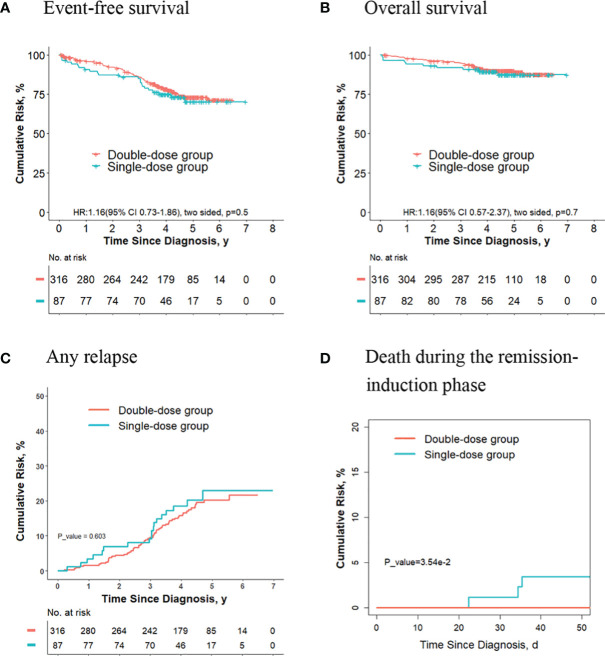
Analysis of survival and cumulative risk of relapse and early death for patients with ALL in final intermediate/high-risk group.

For the 403 patients finally in the intermediate/high-risk group, the 5-year event-free survival rate (73.0% [95%CI, 67.7%-78.8%] vs. 70.2% [95%CI, 60.1%-82.0%]; *P* = 0.50, log-rank test) (**Figure 1A**), the 5-year overall survival rate (89.9% [95%CI, 86.5%-93.4%] vs. 87.4% [95%CI, 80.2%-95.2%]; *P* = 0.70, log-rank test) (**Figure 1B**) and the 5-year cumulative risk of any relapse (20.2% [95%CI, 15.7%-25.2%] vs. 23.0% [95%CI, 13.8%-33.6%]; *P* = 0.60, Gray test) (**Figure 1C**) were not significantly different between the two groups. However, the risk of deaths during the remission-induction phase was significantly lower in the double-dose group (0%) compared with the single-dose group (3.4%; 95%CI, 0.9%-8.9%; *P* = 0.04, Gray test) (**Figure 1D**).

### The Outcomes Are Better for Final Low-Risk Patients Who Receive Two Doses of Daunorubicin Compared With Those Receiving One Dose

**Figure 2:**
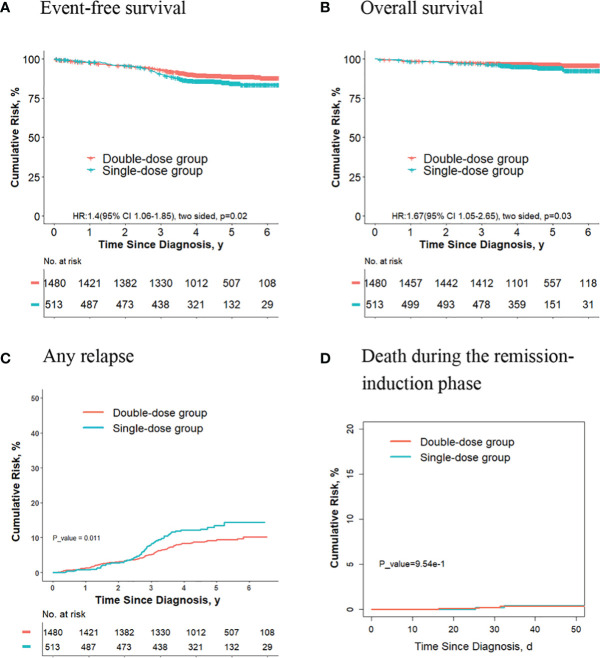
Analysis of survival and cumulative risk of relapse and early death for patients with ALL in the final low-risk group.

For the 1993 final low-risk patients, the 5-year event-free survival rate in the double-dose group (88.8%; 95%CI, 87.1%-90.5%) was significantly better compared with the single-dose group (84.5%; 95%CI, 81.2%-88.1%; *P* = 0.02, log-rank test) (**Figure 2A**). The 5-year overall survival rate was 96.6% (95% CI, 95.6%-97.5%) in the double-dose group, and it was 94.4% (95%CI, 92.2%-96.5%; *P* = 0.03, log-rank test) in the single-dose group (**Figure 2B**). The 5-year cumulative risk of any relapse was significantly lower in the double-dose group (9.4%; 95%CI, 7.9%-11.0%) compared with the single-dose group (16.4%; 95%CI, 10.5%-16.9%; *P* = 0.01, Gray test) (**Figure 2C**). The risk of deaths during the remission-induction phase (0.3% [95%CI, 0.1%-0.8%] *vs*. 0.4% [95%CI, 0.08%-1.3%]; *P* = 0.90, Gray test) was not significantly different between the two groups (**Figure 2D**). There were no significant differences in the cumulative risk of CNS relapse or hematologic relapse between the two groups (data not shown).

**Figure 3:**
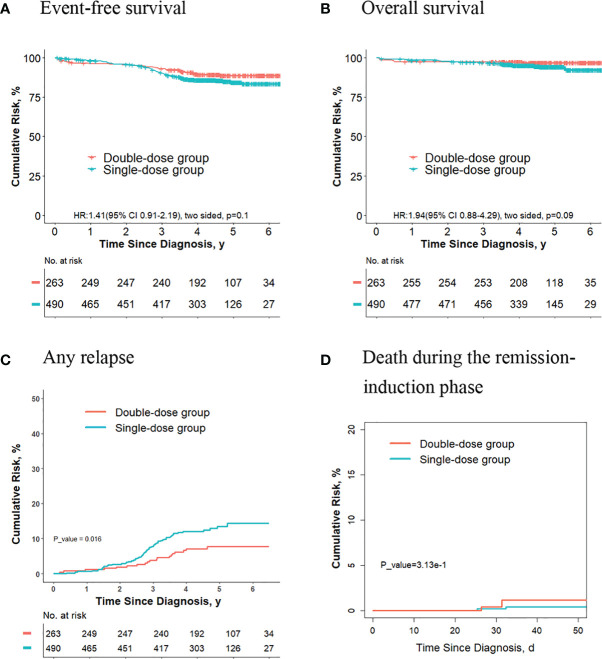
Analysis of survival and cumulative risk of relapse and early death for final low-risk patients with ALL who didn’t meet the criteria for the second dose of daunorubicin.

For the 753 patients who didn’t meet criteria for the second dose of daunorubicin, the 5-year event-free survival rate in the double-dose group (88.7%; 95%CI, 84.7%-92.8%) was not significantly different compared with the single-dose group (84.4%; 95%CI, 80.9%-88.1%; *P* = 0.1, log-rank test) (**Figure 3A**). The 5-year overall survival rate was 96.8% (95% CI, 94.7%-99.0%) in the double-dose group, and it was 94.3% (95%CI, 92.0%-96.5%; *P* = 0.09, log-rank test) in the single-dose group (**Figure 3B**). The 5-year cumulative risk of any relapse was significantly lower in the double-dose group (7.7%; 95%CI, 4.8%-11.6%) compared with the single-dose group (13.5%; 95%CI, 10.4%-17.0%; *P* = 0.016, Gray test) (**Figure 3C**). The risk of deaths during the remission-induction phase (0.7% [95%CI, 0.3%-3.1%] *vs*. 0.3% [95%CI, 0.08%-1.4%]; *P* = 0.30, Gray test) was not significantly different between the two groups (**Figure 3D**).

### No Significant Differences in Outcomes Were Observed for the Final Low-Risk Patients With *ETV6-RUNX1*-Positive ALL or Hyperdiploidy ALL Who Received Two Doses of Daunorubicin Compared With Those Receiving One Dose

Of the 1993 patients eventually assigned to the low-risk group, 656 patients had *ETV6-RUNX1*-positive ALL, and 513 patients had hyperdiploidy ALL. Meanwhile, there were 285 patients with *ETV6-RUNX1*-positive ALL and 218 patients with hyperdiploidy of the 753 children who did not meet the criteria to give second dose of daunorubicin.

**Figure 4:**
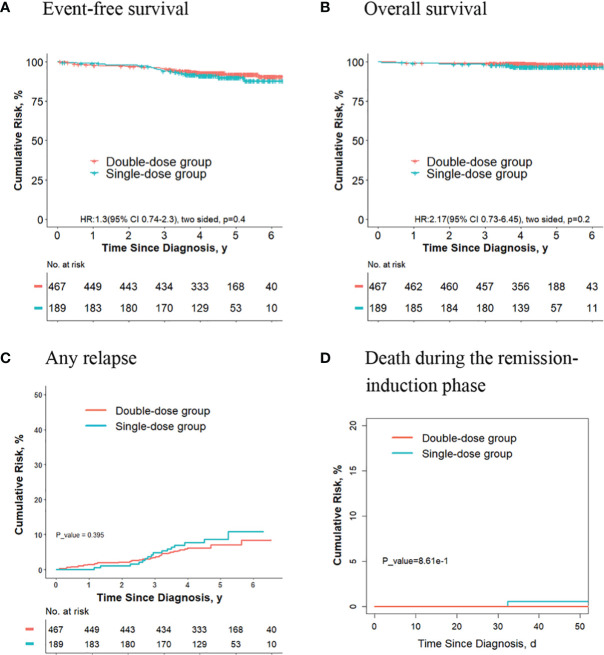
Analysis of survival and cumulative risk of relapse and early death for final low-risk patients with *ETV6-RUNX1*-positive ALL.

For final low-risk patients with *ETV6-RUNX1*-positive ALL, there were no significant differences in terms of the 5-year event-free survival rate (90.0% [95%CI, 85.5%-94.7%] vs. 91.9% [95%CI, 89.2%-94.6%]; *P* = 0.30, log-rank test) (**Figure 4A**), the 5-year overall survival rate (96.6% [95%CI, 94.0%-99.3%] vs. 98.3% [95%CI, 97.0%-99.6%]; *P* = 0.20, log-rank test) (**Figure 4B**), the 5-year cumulative risk of any relapse (8.7% [95%CI, 5.0%-13.6%] vs. 7.1% [95%CI, 4.8%-9.9%]; *P* = 0.40, Gray test) (**Figure 4C**), and the risk of deaths during the remission-induction phase (0.5% [95%CI, 0.05%-2.7%] vs. 0%; *P* = 0.90, Gray test) (**Figure 4D**) between the single-dose group (189 patients) and double-dose group (467 patients).

**Figure 5:**
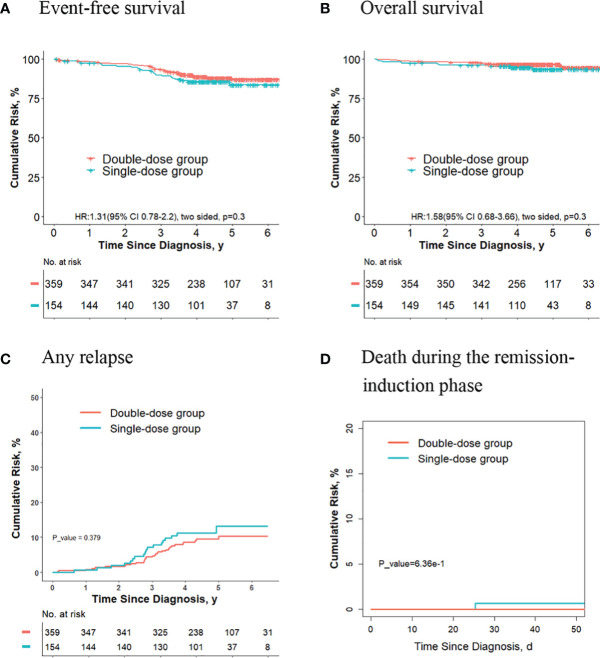
Analysis of survival and cumulative risk of relapse and early death for final low-risk patients with hyperdiploidy ALL.

For final low-risk patients with hyperdiploidy ALL, no significant differences were observed in terms of the 5-year event-free survival rate (83.6% [95%CI, 77.0%-90.7%] vs. 87.2% [95%CI, 83.4%-91.1%]; *P* = 0.30, log-rank test) (**Figure 5A**), the 5-year overall survival rate (93.5% [95%CI, 89.3%-97.8%] vs. 96.6% [95%CI, 94.8%-98.5%]; *P* = 0.30, log-rank test) (**Figure 5B**), the 5-year cumulative risk of any relapse (13.1% [95%CI, 7.7%-20.0%] vs. 10.3% [95%CI, 7.1%-14.1%]; *P* = 0.40, Gray test) (**Figure 5C**), and the risk of deaths during the remission-induction phase (0.6% [95%CI, 0.1%-3.3%] vs. 0%; *P* = 0.60, Gray test) (**Figure 5D**) between the single-dose group (154 patients) and double-dose group (359 patients).

We also compared the outcomes between the two groups for final low-risk patients with *ETV6-RUNX1*-positive ALL or hyperdiploidy ALL who did not meet the criteria to give second dose of daunorubicin, and no significant differences were observed in terms of the 5-year event-free survival rate, the 5-year overall survival rate, the 5-year cumulative risk of any relapse, and the risk of deaths during the remission-induction phase (**Supplementary Figures 1, 2**).

### The Outcomes Are Significantly Better for Final Low-Risk Patients Without *ETV6-RUNX1* Positivity and Hyperdiploidy ALL Who Received Two Doses of Daunorubicin Compared With Those Receiving One Dose

**Figure 6:**
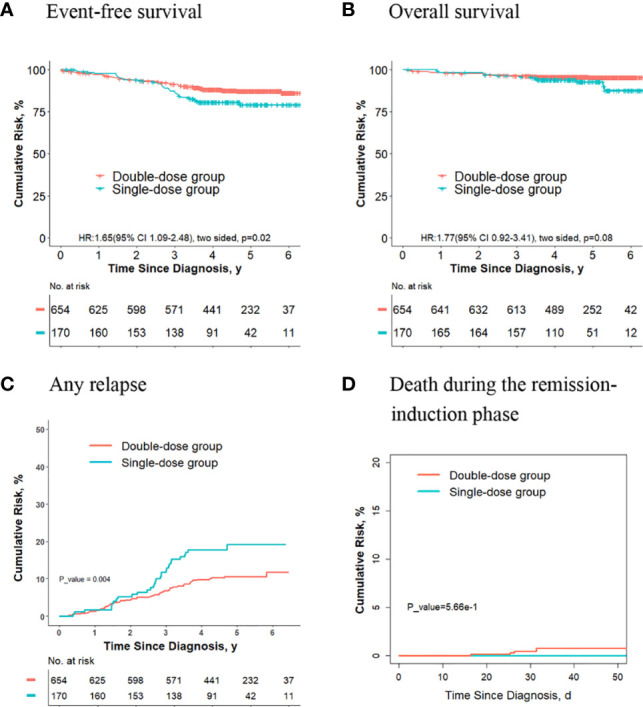
Analysis of survival and cumulative risk of relapse and early death for final low-risk patients without *ETV6-RUNX1* positivity or hyperdiploidy ALL.

Of the other 824 patients, who had neither *ETV6-RUNX1*-positive ALL nor hyperdiploidy ALL, 170 received one dose of daunorubicin, and 654 received two doses. For these patients, the 5-year event-free survival rate in the double-dose group (87.4%; 95%CI, 84.8%-90.1%) was significantly better compared with the single-dose group (79.1%; 95%CI, 72.7%-86.1%; *P* = 0.02, log-rank test) (**Figure 6A**). The 5-year overall survival rate was 95.3% (95% CI, 93.6%-97.0%) in the double-dose group, and it was 92.7% (95%CI, 88.5%-97.1%; *P* = 0.08, log-rank test) in the single-dose group (**Figure 6B**). The 5-year cumulative risk of any relapse was significantly lower in the double-dose group (10.5%; 95%CI, 8.3%-13.1%) compared with the single-dose group (19.2%; 95%CI, 13.3%-26%; *P* = 0.004, Gray test) (**Figure 6C**). The risk of deaths during the remission-induction phase (0.8% [95%CI, 0.3%-1.7%] *vs*. 0%; *P* = 0.6, Gray test) was not significantly different between the two groups (**Figure 6D**).

**Figure 7:**
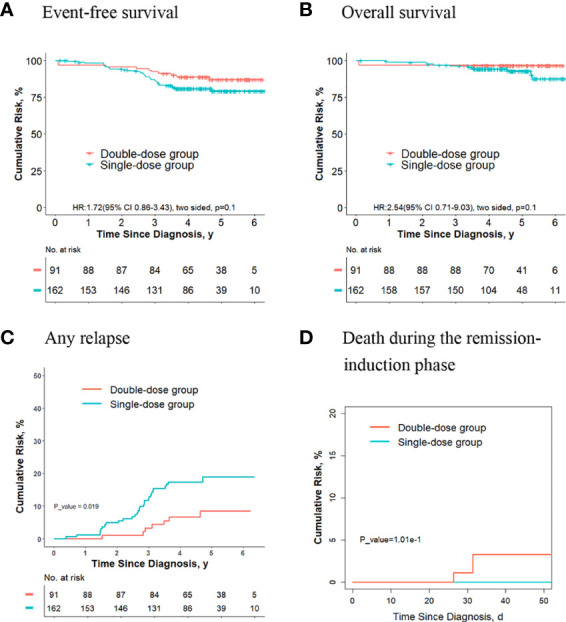
Analysis of survival and cumulative risk of relapse and early death for final low-risk patients without *ETV6-RUNX1* positivity or hyperdiploidy ALL and didn’t meet the criteria for the second dose of daunorubicin.

Of the 824 patients, 253 didn’t meet criteria for the second dose of daunorubicin. For these patients, no significant differences were observed in terms of the 5-year event-free survival rate (87.1% [95%CI, 80.1%-94.7%] vs. 79.2% [95%CI, 72.6%-86.5%]; *P* = 0. 01, log-rank test) (**Figure 7A**), the 5-year overall survival rate (96.7% [95%CI, 93.1%-99.9%] vs. 87.5% [95%CI, 79.5%-96.3%]; *P* = 0. 1, log-rank test) (**Figure 7B**) and the risk of deaths during the remission-induction phase (3.3% [95%CI, 0.9%-8.6%] vs. 0%; *P* = 0.1, Gray test) (**Figure 7D**) between the two groups, however, the 5-year cumulative risk of any relapse (8.5% [95%CI, (3.6%-16.1%)] vs. 19.0% [95%CI, 13.0%-26.0%]; *P* = 0.019, Gray test) was significantly lower in the double-dose group compared with the single-dose group (**Figure 7C**).

## Discussion

The chemotherapy intensity for pediatric ALL treatment has been raised to the limit of tolerance and can no longer be “pushed” to obtain improved results (24). How to reduce the adverse effects of chemotherapy to improve the quality of life while maintaining or even improving their cure high rates for children with ALL has drawn much attention from the researchers. It is a feasible way to reduce the treatment dose intensity of conventional chemotherapy for low-risk patients. This retrospective study of a large sample of patients provided clinical evidence for reducing the intensity of chemotherapy in children with low-risk ALL and gave a sound basis for the design of prospective randomized controlled studies or modification of the protocol in the future.

Several ALL cooperative groups have strived for an attempt that patients with the favorable clinical outcome can be spared from more intensive and toxic treatment (8, 25–27). The SCMC has reported over 10 years ago that patients in the low-risk group treated with a reduced dosage of daunorubicin during the induction phase can achieve better results, with the remission rate at 95%, while the infection rate goes down, and the infection-related mortality rate is reduced to 0 (28). Moreover, some chemotherapy regimens, such as Dutch Childhood Leukemia Study Group protocol (Dutch ALLV) (29), Children’s Cancer Group (CCG)1952 study (30) and UKALL XI from United Kingdom Medical Research Council (UKMRC) (31), which did not include daunorubicin during remission-induction therapy, are also able to achieve a remission rate of more than 95%. Total XV study demonstrated that prophylactic cranial irradiation can be totally omitted without compromising overall survival, relying on intensive dexamethasone, vincristine and asparaginase, high-dose methotrexate, and, for patients with blasts in cerebrospinal fluid, extra doses of intrathecal therapy (9). By retrospectively analyzing the clinical data of the Guangdong (GD)-2008-ALL collaborative group, the protocol of which was carried out based on Berlin-Frankfurt-Münster (BFM) 2002 backbone treatment, the researchers found that reduced intensity of early intensification does not increase the risk of relapse in children with standard risk acute lymphoblastic leukemia (32). In a recent study (Recife RELLA05 pilot study), the researchers demonstrated that long-term survival rates exceeding 90% could be obtained in ~25% of children with B-ALL treated with a mildly myelosuppressive chemotherapy regimen (33). These findings led us to propose that children with ALL with favorable presenting features could be selected to receive reduced–dose intensity treatment regimens.

In the CCCG-ALL-2015 protocol, the standard remission induction therapy includes two doses of daunorubicin on day 5 and day 12 for low-risk patients. In actual clinical practice, however, some patients receive the second dose of daunorubicin, while some do not because of their ineligible hematopoietic recovery or physical conditions. In the present study, therefore, we investigated whether the second dose of daunorubicin in the induced remission phase could be omitted for low-risk patients.

Considering that patients in the low-risk group and intermediate/high-risk groups received chemotherapy regimens with different intensities, we compared them separately by risk groups. Up to the follow-up day, there were almost no significant differences in outcomes between the single-dose group and double-dose group for final intermediate/high-risk patients, probably because they received more intense chemotherapy from the consolidation phase. However, for final low-risk patients, those in the double-dose group had better outcomes compared with the single-dose group in terms of the 5-year event-free survival rate and the 5-year cumulative risk of any relapse, though the 5-year overall survival rate and the risk of early deaths were not significantly different between the two groups. It appeared that the second dose of daunorubicin should not be omitted in terms of long-term prognosis.

However, should the second dose of daunorubicin be given for all patients in the low-risk group? In another study, patients age 1 to 17 years with low-risk B-ALL, defined by the absence of high-risk genetic features (*BCR-ABL1, KMT2A-AFF1*) and the MRD level < 1 × 10^–4^ on days 33 and 78, are randomized to receive standard or reduced delayed intensification treatment (34). This modification results in a poorer overall 8-year disease-free survival and overall survival, except that the patients with *ETV6-RUNX1*-positive ALL or age of 1 to 6 years are equally well in both treatment arms. This study shows that treatment reduction in this context is only feasible in specific subgroups of patients with low-risk ALL.

Two of the most common subtypes of pediatric ALL, *ETV6-RUNX1* fusion and hyperdiploidy, are both associated with a favorable prognosis (35–37). In St. Jude Total Therapy Study XV, only low-risk B-ALL patients with *ETV6-RUNX1* positivity or hyperdiploidy and negative MRD (<10^–4^) on day 19 of remission induction have a low cumulative risk of relapse (1.9% and 3.8%, respectively) compared to an unacceptably high cumulative risk of relapse (9.5%) in low-risk patients with other genotypes and negative MRD on day 19 (38). Therefore, patients with *ETV6-RUNX1*-positive or hyperdiploidy ALL and others were analyzed separately. The results are as expected. First, there were no differences in outcome for patients with *ETV6-RUNX1*-positive or hyperdiploidy ALL between the two groups. Second, other patients in the double-dose group had better outcomes compared with the single-dose group, particularly in terms of EFS and relapse. Therefore, the second dose daunorubicin in remission induction should not be omitted in all B-ALL patients, except those with *ETV6-RUNX1* positivity or hyperdiploidy. In our study, 824 patients eventually assigned to the low-risk group had neither *ETV6-RUNX1*-positive ALL nor hyperdiploidy ALL. Of these patients, 170 required two doses of daunorubicin but received only one dose actually. Therefore, 170 of these patients required dose modifications in chemotherapy regimen.

Ten patients died from complications such as infection or bleeding related to bone marrow suppression during remission induction, with 5 receiving one dose and 5 receiving two doses of daunorubicin. Therefore, the treatment related mortality in patients receiving higher and low dose were 0.28% (5/1796) and 0.83% (5/600), respectively. However, we attributed these deaths to the combination of multiple chemotherapeutic agents, not just daunorubicin. We speculated that the deaths were closely related to the severity of bone marrow suppression.

This study has two main limitations. First of all, this is not a prospective randomized controlled study. The information of daunorubicin administration was retrospectively collected, which might not be complete and with recall bias and subjective bias. Second, we mainly selected children with low WBC number or ANC as the research objects, which could not prove that children with high WBC number could also reduce the dose of daunorubicin. However, our results might provide a basis for design of prospective randomized controlled studies or modification of protocol in the future.

In conclusion, our results suggested that only B-ALL patients with a favorable genotype (*ETV6-RUNX1* positivity or hyperdiploidy) who achieved an early negative MRD status (10^–4^) were suitable candidates for dosage reduction of daunorubicin during the induction remission. It is worth mentioning that the CCCG-ALL-2020 protocol, which has been started in July 2020, has been modified based on the results of our study. Therefore, all B-ALL patients, except those with *ETV6-RUNX1* positivity or hyperdiploidy, will receive two doses of daunorubicin during induction remission. If the WBC counts are low (WBC <1×10^9^/L or ANC<0.3×10^9^/L) on day 12, the second dose will be postponeed but not omitted. Collectively, chemotherapy regimens need to be further optimized to minimize chemotherapy and reduce side effects for ALL patients with favorable presenting features on the premise of ensuring the therapeutic effect.

## Data Availability Statement

The original contributions presented in the study are included in the article/**Supplementary Material**. Further inquiries can be directed to the corresponding authors.

## Ethics Statement

The studies involving human participants were reviewed and approved by the ethics committee of each participating institution. Written informed consent to participate in this study was provided by the participants’ legal guardian/next of kin.

## Author Contributions

XJ and JY were leading principal investigators of this study, contributed equally to study conception and design, data analysis and interpretation, manuscript writing, revise and editing. XFZ, SS, JY, SH, JG, HJ, XJ, XWZ, XT, YF, RJ, QH, HuiJ, NW, LS, WL, MY, KP, XW and CL were involved in patient recruitments and data acquisition. YZ, KW, JC, and SS contributed to data acquisition and data analysis. All authors read and approved the final manuscript.

## Funding

This study was supported by the VIVA China Children’s Cancer Foundation, and funded by Clinical Research Center of Shandong University (No. 2020SDUCRCA010).

## Conflict of Interest

The authors declare that the research was conducted in the absence of any commercial or financial relationships that could be construed as a potential conflict of interest.

## Publisher’s Note

All claims expressed in this article are solely those of the authors and do not necessarily represent those of their affiliated organizations, or those of the publisher, the editors and the reviewers. Any product that may be evaluated in this article, or claim that may be made by its manufacturer, is not guaranteed or endorsed by the publisher.
