# Supplementary material for: Reduced Dose Intensity of Daunorubicin During Remission Induction for Low-Risk Patients With Acute Lymphoblastic Leukemia: A Retrospective Cohort Study of the Chinese Children’s Cancer Group
Source: Front Oncol. 2022 Jun 7;12:911567. doi: 10.3389/fonc.2022.911567 (PMC9209708; doi:10.3389/fonc.2022.911567)
Supplement: Supplementary file 1 [file DataSheet_1.docx]

Supplementary Material

# Supplementary Figures


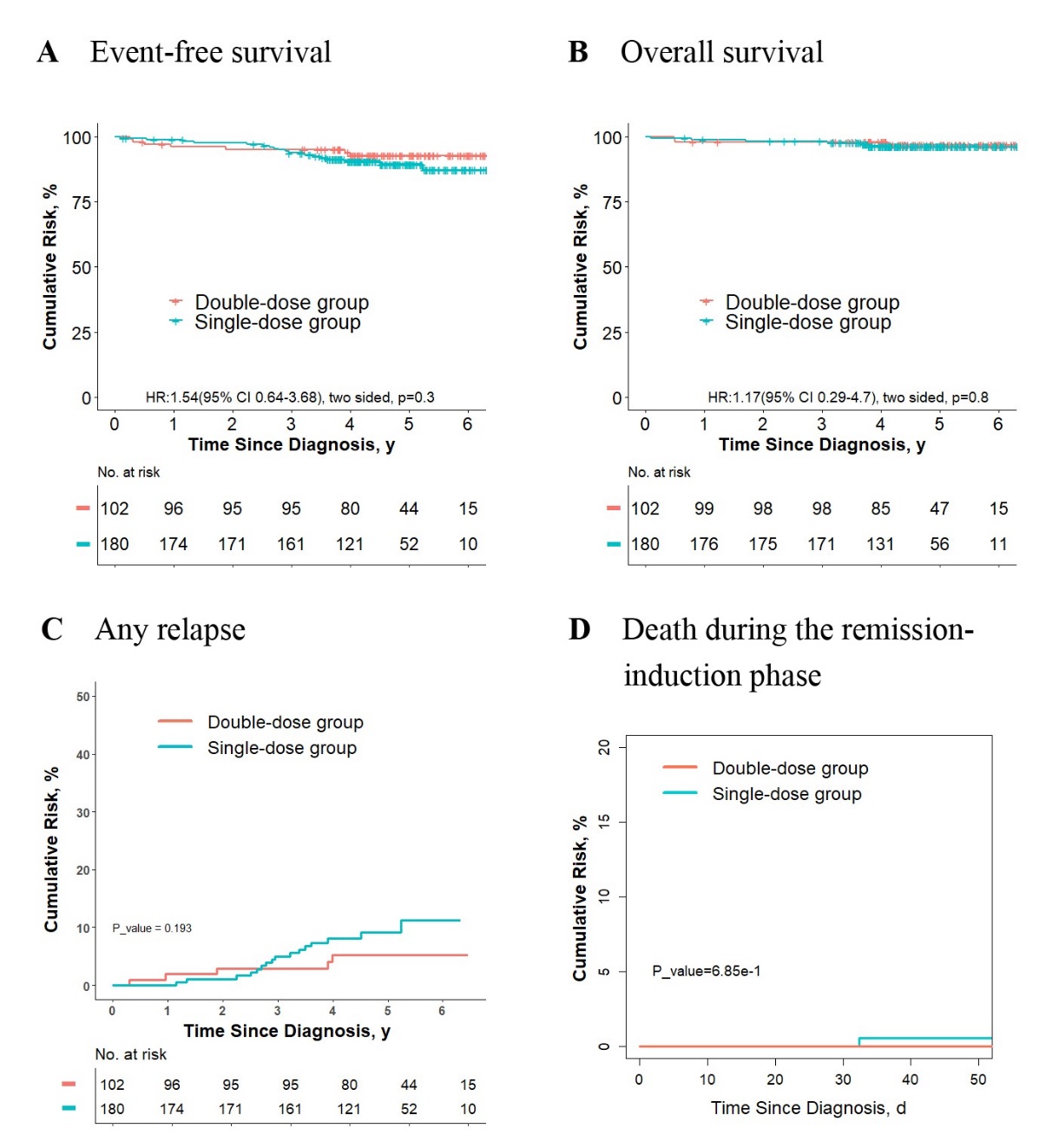


**Supplementary Figure 1.** Analysis of survival and cumulative risk of relapse and early death for final low-risk patients with *ETV6-RUNX1*-positive ALL who didn’t meet the criteria for the second dose of daunorubicin.


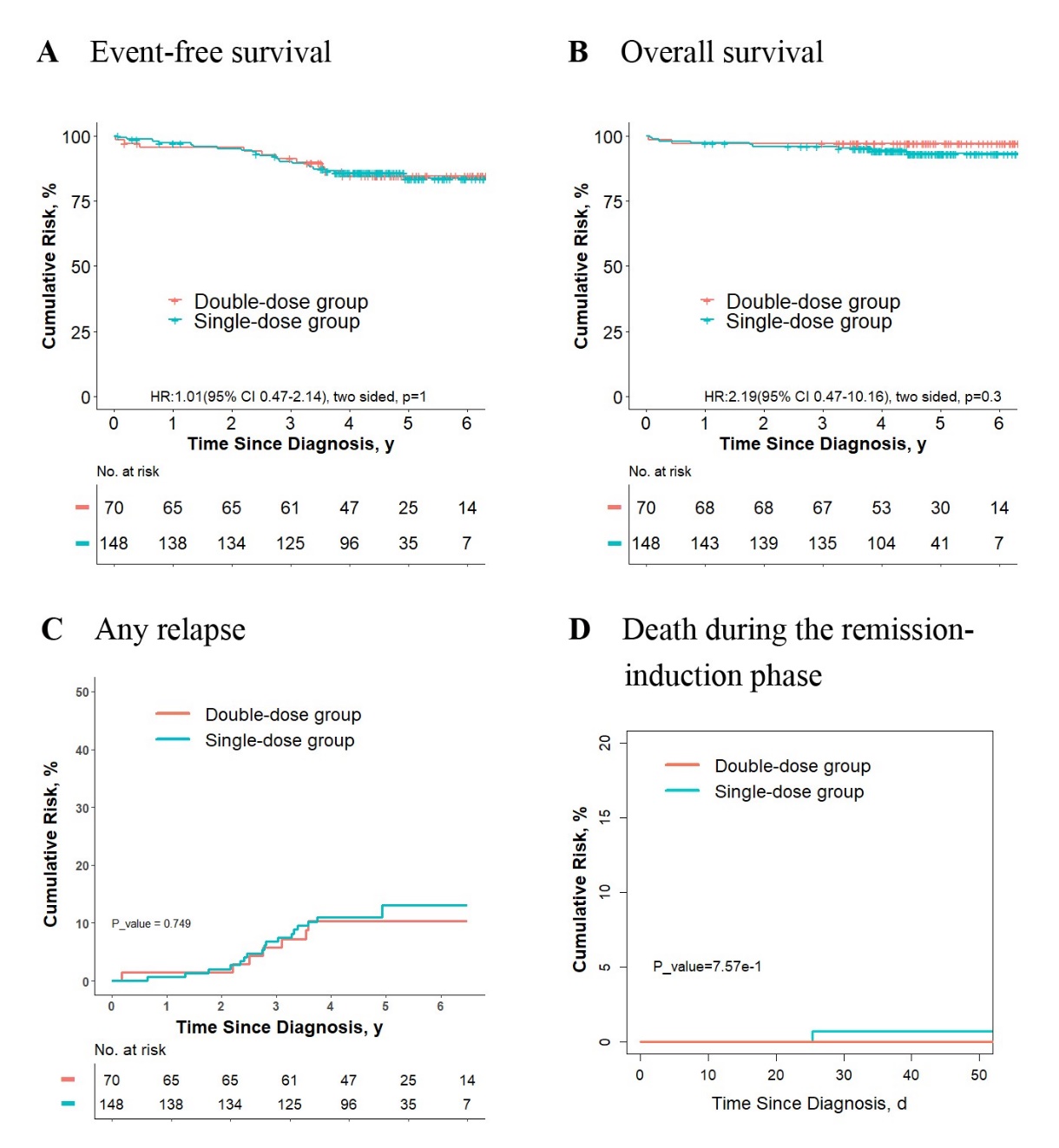


**Supplementary Figure 2.** Analysis of survival and cumulative risk of relapse and early death for final low-risk patients with hyperdiploidy ALL who didn’t meet the criteria for the second dose of daunorubicin.
